# Supplementary material for: Time-series transcriptome analysis identified differentially expressed genes in broiler chicken infected with mixed Eimeria species
Source: Front Genet. 2022 Aug 8;13:886781. doi: 10.3389/fgene.2022.886781 (PMC9393255; doi:10.3389/fgene.2022.886781)
Supplement: Supplementary file 2 [file DataSheet1.ZIP › 4dpi_KEGG.Gsea.1625100298584/KEGG_GLYCOSPHINGOLIPID_BIOSYNTHESIS_LACTO_AND_NEOLACTO_SERIES.html]

Details for gene set KEGG\_GLYCOSPHINGOLIPID\_BIOSYNTHESIS\_LACTO\_AND\_NEOLACTO\_SERIES[GSEA]

|  || Dataset | TMM\_4dpi\_gct\_format\_4dpi\_gct\_format.Class\_4dpi.cls #PC\_versus\_NC.Class\_4dpi.cls #PC\_versus\_NC\_repos |
| Phenotype | Class\_4dpi.cls#PC\_versus\_NC\_repos |
| Upregulated in class | 1 |
| GeneSet | KEGG\_GLYCOSPHINGOLIPID\_BIOSYNTHESIS\_LACTO\_AND\_NEOLACTO\_SERIES |
| Enrichment Score (ES) | 0.5602068 |
| Normalized Enrichment Score (NES) | 1.52961 |
| Nominal p-value | 0.044857766 |
| FDR q-value | 0.28887665 |
| FWER p-Value | 0.9418 |
Table: GSEA Results Summary

  

Fig 1: Enrichment plot: KEGG\_GLYCOSPHINGOLIPID\_BIOSYNTHESIS\_LACTO\_AND\_NEOLACTO\_SERIES      
 Profile of the Running ES Score & Positions of GeneSet Members on the Rank Ordered List

  

| SYMBOL | TITLE | RANK IN GENE LIST | RANK METRIC SCORE | RUNNING ES | CORE ENRICHMENT || 1 | FUT9 | na | 20 | 2.261 | 0.2645 | Yes |
| 2 | B3GALT5 | na | 110 | 1.594 | 0.4448 | Yes |
| 3 | B3GNT5 | na | 331 | 1.136 | 0.5602 | Yes |
| 4 | B3GNT3 | na | 2588 | 0.337 | 0.4118 | No |
| 5 | B4GALT4 | na | 2748 | 0.315 | 0.4355 | No |
| 6 | B3GALT2 | na | 3268 | 0.247 | 0.4213 | No |
| 7 | ST3GAL3 | na | 4490 | 0.125 | 0.3342 | No |
| 8 | FUT6 | na | 4700 | 0.106 | 0.3293 | No |
| 9 | B4GALT1 | na | 5657 | 0.020 | 0.2520 | No |
| 10 | GCNT2 | na | 6222 | -0.024 | 0.2079 | No |
| 11 | ST8SIA1 | na | 6635 | -0.057 | 0.1802 | No |
| 12 | FUT4 | na | 7683 | -0.151 | 0.1107 | No |
| 13 | B4GALT2 | na | 7886 | -0.169 | 0.1138 | No |
| 14 | ST3GAL6 | na | 9410 | -0.338 | 0.0266 | No |
| 15 | B3GNT2 | na | 9925 | -0.402 | 0.0310 | No |
| 16 | B4GALT3 | na | 10599 | -0.519 | 0.0359 | No |
| 17 | FUT7 | na | 11274 | -0.693 | 0.0614 | No |
Table: GSEA details [plain text format]

  

Fig 2: KEGG\_GLYCOSPHINGOLIPID\_BIOSYNTHESIS\_LACTO\_AND\_NEOLACTO\_SERIES      
 Blue-Pink O' Gram in the Space of the Analyzed GeneSet

  

Fig 3: KEGG\_GLYCOSPHINGOLIPID\_BIOSYNTHESIS\_LACTO\_AND\_NEOLACTO\_SERIES: Random ES distribution      
 Gene set null distribution of ES for **KEGG\_GLYCOSPHINGOLIPID\_BIOSYNTHESIS\_LACTO\_AND\_NEOLACTO\_SERIES**
